# Podoplanin Drives Motility of Active Macrophage *via* Regulating Filamin C During *Helicobacter pylori* Infection

**DOI:** 10.3389/fimmu.2021.702156

**Published:** 2021-10-11

**Authors:** Yi Ying Cheok, Grace Min Yi Tan, Keith Conrad Fernandez, Yee Teng Chan, Chalystha Yie Qin Lee, Heng Choon Cheong, Chung Yeng Looi, Jamuna Vadivelu, Suhailah Abdullah, Won Fen Wong

**Affiliations:** ^1^ Department of Medical Microbiology, Faculty of Medicine, University of Malaya, Kuala Lumpur, Malaysia; ^2^ School of Bioscience, Taylor’s University, Subang Jaya, Selangor, Malaysia; ^3^ Department of Medicine, Faculty of Medicine, University of Malaya, Kuala Lumpur, Malaysia

**Keywords:** podoplanin, macrophage, *Helicobacter pylori*, cell migration, Filamin C, interleukin-1β

## Abstract

Podoplanin (Pdpn) is a mucin-type transmembrane protein that has been implicated in multiple physiological settings including lymphangiogenesis, platelet aggregation, and cancer metastasis. Here, we reported an absence of Pdpn transcript expression in the resting mouse monocytic macrophages, RAW264.7 cells; intriguingly, a substantial upregulation of Pdpn was observed in activated macrophages following *Helicobacter pylori* or lipopolysaccharide stimulation. Pdpn-knockout macrophages demonstrated intact phagocytic and intracellular bactericidal activities comparable to wild type but exhibited impaired migration due to attenuated filopodia formation. In contrast, an ectopic expression of Pdpn augmented filopodia protrusion in activated macrophages. NanoString analysis uncovered a close dependency of Filamin C gene on the presence of Pdpn, highlighting an involvement of Filamin C in modulation of actin polymerization activity, which controls cell filopodia formation and migration. In addition, interleukin-1β production was significantly declined in the absence of Pdpn, suggesting a role of Pdpn in orchestrating inflammation during *H. pylori* infection besides cellular migration. Together, our findings unravel the Pdpn network that modulates movement of active macrophages.

## Introduction

Podoplanin is a 36- to 43-kDa mucin-type transmembrane *O*-glycoprotein that is predominantly expressed on the surface of lymphatic endothelial cells, kidney podocytes, and lung type I alveolar cells (1–3). This glycoprotein has multiple physiological functions such as lymphangiogenesis (4, 5), heart and lung organogenesis (6, 7), platelet aggregation (8), and epithelial–mesenchymal transition (EMT) (9, 10). Given its broad functions, *Pdpn*-null mice are embryonically lethal due to impaired lymphatic development (11), cardiac anomalies, and respiratory failure caused by abnormal heart and lung morphogenesis (6, 7).

Current knowledge of Pdpn functions relies heavily on the extracellular domain interaction with its ligand platelet C-type lectin-like receptor 2 (Clec2). Interaction of Pdpn to Clec2 stimulates deep vein thrombosis (12) while point mutations of threonine residues at the highly conserved platelet-activating (PLAG) domain of Pdpn obliterate platelet aggregation (13). By activating platelets, Pdpn is able to promote pulmonary cancer growth, malignant progression, and metastasis through cancer embolization that protects cancer cells from immunological assault (14, 15). In addition, Pdpn–Clec2 axis controls the development and maintains the integrity and contractibility of high endothelial venules (HEVs) (4, 5, 11), permitting the travel of lymphocytes and dendritic cells across the lymphatic system (16, 17). While the extracellular domain of Pdpn binds to Clec2, the intracellular domain of Pdpn interacts with the ezrin–radixin–moesin (ERM) complex to modulate Rho-GTPases and actin cytoskeletal rearrangement (10, 18). High expression of Pdpn has been associated with cancer metastasis such as advanced-stage gastric carcinoma (15, 19). On the contrary, Pdpn inhibition by neutralizing antibody depresses the metastasis of breast cancer cells xenografted into immunodeficient NOD-SCID mouse model (20). As such, Pdpn has hitherto been considered a potential marker as well as therapeutic target for aggressive cancer (21, 22).

In the immune system, *Pdpn* expression has been reported in T helper 17 (T_H_17) cells and macrophages (23, 24). *Pdpn*-expressing T_H_17 cell promotes ectopic lymphoid follicles formation in mouse experimental autoimmune encephalomyelitis (EAE) model (23), while its deletion exacerbates EAE in a genetically susceptible mouse model (25). In macrophages, *Pdpn* expression can be induced by thioglycollate or lipopolysaccharide (LPS) (24). A subset of *Pdpn*-expressing macrophage is highly phagocytic (26), associates with pro-inflammatory response (27), and promotes platelet aggregation through Pdpn–Clec2 crosstalk (24). Presence of *Pdpn*-expressing CD68^+^ macrophage-like cells and synovial fibroblasts at the joint of rheumatoid arthritis patients exaggerate joint cartilage inflammation and destruction (28, 29). Inflammatory response can be relieved with *Vav1*-promoter driven *Pdpn* deletion in myeloid cells as reported in mouse peritonitis model (30).

Chronic inflammation triggered by long-term *Helicobacter pylori* infection is the major mechanism underlying the development of gastric cancer in human. Macrophage is a key player in orchestrating inflammatory response to *H. pylori* infection since increased M1 cells and related pro-inflammatory markers were detected in the stomach of *H. pylori*-infected humans (31–34), while transient macrophage depletion reduces the *H. pylori*-associated inflammation and gastric pathology in a mouse model (35). While T_H_17 cells also play an important role during the chronic inflammation induced by *H. pylori*, the current study focuses on the role of Pdpn in macrophages only. This study investigates the expression and potential role of Pdpn in macrophage during inflammation by applying the *H. pylori* infection model. Subsequent generation of *Pdpn*-knockout and overexpressing macrophages enabled us to clarify the function and signaling pathway of Pdpn. Here, we demonstrated that, during *H. pylori* infection, Pdpn promotes *Filamin C* expression to enhance filopodia formation and migratory activity of macrophages. Nonetheless, there are no differences in bacterial uptake and killing in the absence of Pdpn. High *Pdpn* expression is also associated with a greater IL-1β secretion, which implies intervening Pdpn as a therapeutic opportunity in limiting inflammation.

## Materials and Methods

### Bacteria Strains and Infection Models

*H. pylori* Sydney Strain 1 (SS1) was provided by the *Helicobacter pylori* Research Laboratory, University of Western Australia. *H. pylori* J99 strain (700824), *Neisseria gonorrhoeae* (BAA-1737), *Staphylococcus aureus* (BAA-811), and *S. epidermidis* (BAA-35984) were purchased from the American Type Culture Collection (ATCC). *H. pylori* and *N. gonorrhoeae* strains were grown on chocolate agar plate supplemented with 7% laked horse blood (Oxoid, Basingstoke, UK) under microaerophilic conditions at 10% CO_2_, 37°C in a humidified incubator and were sub-cultured every 3 days. *S. aureus* and *S. epidermidis* were cultured on nutrient agar at 37°C in a humidified incubator.

RAW264.7 (ATCC^®^ TIB-71™) and human THP1 (ATCC^®^ TIB-202™) macrophages were purchased from the ATCC. RAW264.7 cells were cultured in DMEM supplemented with 10% heat-inactivated FBS at 37°C, 5% CO_2_. THP-1 cells were cultured in RPMI 1640 medium supplemented with 10% FBS, 10 mM HEPES (pH 7.4), 1 mM sodium pyruvate, 1× non-essential amino acids, and 50 μM 2-mercaptoethanol. RAW264.7 cells were seeded 1 day prior to infection, while THP-1 cells were seeded immediately before infection, at 5 × 10^5^ cells/ml. Freshly cultured bacteria were harvested in brain heart infusion (BHI) broth, measured by a spectrophotometer (OD_650nm_ of 1 = 1 × 10^8^ cells/ml) and infected at MOI of 1, 5 or 10.

### CRISPR/Cas9 Gene Editing-Mediated Pdpn Deletion

*Pdpn*-KD/TC and *Pdpn*-KO clones were generated using CRISPR/Cas9 gene editing tool. CRISPR/Cas9 target site (5’-ACAACCACAGGTGCTACTGG-3’) was selected from exon 2 of mouse *Pdpn* gene and cloned into GeneArt^®^ CRISPR Nuclease (OFP) vector (Thermo Fisher Scientific, Waltham, MA) with an orange fluorescence protein reporter. A total of 500 ng of plasmid was added to 1.5 μl Lipofectamine 3000 (Thermo Fisher Scientific) and transfected into 2 × 10^5^ cells/ml RAW264.7 cells. OFP^+^ cells were sorted individually into 96-well plates using FACSAria III (BD Bioscience), grown at 37°C for 14 days, and DNA was isolated from each clone using phenol chloroform method as previously described (36). CRISPR target site was amplified using two flanking primers (5’-AGGCTCCAACGAGATCAAGA-3’ and 5’-AGCTCTTTAGGGCGAGAACC-3’) and sequenced. Sequencing data were analyzed using BioEdit (version 7.2.5) and aligned with European Bioinformatics Institute (EMBL-EBI) clustalW2 multiple sequence alignment system.

### Establishment of Pdpn Overexpression Model Using Retrovirus Transduction System

*Pdpn* cDNA was amplified from *H. pylori*-stimulated RAW264.7 cells with KAPA HiFi PCR Kit (KAPA Biosystems, Wilmington, MA). using 5’-CGGAATTCTGGACCGTGCCAGTGTTGTTCTG-3’ and 5’-GGGATATCTTAGGGCGAGAACCTTCCAGAAATC-3’ primers. *Eco*RI and *Eco*RV recognition sequences (underlined sequence) were added to ease cloning into pcDNA3.1 vector. Reference sequence was obtained from the National Center for Biotechnology Information (NCBI Accession No. NM_010329.3). *Pdpn* cDNA were subsequently cut from pcDNA3.1 vector and inserted into retroviral vector (pMX-IRES-GFP) using *Eco*RI and *Xho*I enzymes. A mixture of 2.5 μg of plasmid and 3.75 μl of Lipofectamine 3000 (Thermo Fisher Scientific) were transfected into 3 × 10^5^ cells/ml PLAT-E retroviral packaging cells (Cell Biolabs, San Diego, CA) in a six-well plate. After 48 h, retroviral supernatant was harvested, mixed with fresh medium at ratio 1:1 with 8 μg/ml polybrene, and transduced in 2 × 10^5^ cells/ml RAW264.7 cells. GFP^+^ cells were sorted using FACSAria III (BD Bioscience) individually into 96-well plate, grown for 14 days to form a colony of approximately 10^5^ cells.

### Microarray and Quantitative RT-PCR

Microarray data were performed with Agilent Technologies microarray platform using Agilent Sure Print G3 Human GE 8 (Design ID: G4851A, Lot: 0006097429) as previously described (37). Microarray data are available at https://www.ncbi.nlm.nih.gov/geo/query/acc.cgi?acc=GSE151289. RNA was isolated using TRIzol reagent (Invitrogen, Carlsbad, CA) as previously described (38). cDNA was prepared using Moloney murine leukemia virus (MMLV) reverse transcriptase (Invitrogen). Quantitative RT-PCR was performed using SsoAdvanced SYBR Green Supermix (Biorad, Hercules, CA) in Strategene Mx3000P qPCR system (Agilent, Santa Clara, CA). Endogenous *Pdpn* was amplified using primers 5’-AGGCTCCAACGAGATCAAGA-3’ and 5’-AGCTCTTTAGGGCGAGAACC-3’, while both exogenous and endogenous *Pdpn* were examined using primers 5’-TTT GGGGAGCGTTTGGTTCT-3’ and 5’-GCAAGCCATCTCTATTGGGGT-3’. *β-actin* primers (5’-GATGACGATATCGCTGCGCTG-3’ and 5’-GTACGACCAGAGGCATACAGG-3’) were used as a control. Relative fold changes were calculated using 2^−ΔΔCT^ formula.

### Immunoblot Analysis

Cells were lysed in RIPA lysis buffer supplemented with protease inhibitor cocktail (1:1,000), 200 µm PMSF, and 100 µM sodium orthovanadate (Santa Cruz Biotech, Santa Cruz, CA), loaded and separated by SDS-PAGE, before blotted onto polyvinylidene fluoride (PVDF) membranes. Membranes were blocked with 5% BSA in Tris buffered saline-10% Tween 20 (TBS-T) and incubated with primary (1:1,000) and secondary (1:5,000) antibodies. Primary antibodies used were anti-β-actin (Cell Signaling Technologies, Beverly, MA), anti-Pdpn (R&D Systems, Minneapolis, MN), biotin anti-human Pdpn (clone NC-08) (BioLegend), and anti-Filamin C (Novus Biologicals, USA) antibodies. Secondary antibodies used were alkaline phosphatase-conjugated rabbit or goat anti-IgG antibodies (Promega, Madison, WI). Membranes were developed using nitro blue tetrazolium/5-bromo-4-chloro-3-indolyl phosphate (NBT-BCIP) substrate.

### Phagocytosis Assay

*H. pylori* (10^8^ cells/ml) were labeled with FITC in the dark for 30 min and washed 4× with sterile PBS. Cells were infected with FITC-labeled bacteria at MOI 1 for 1 and 4 h before analyzed. Cells harvested at 1 and 4 h were washed and analyzed with FACS Canto II (BD Bioscience). For immunofluorescent staining, cells were seeded on a coverslip and infected with *H. pylori* at MOI 10. At 24 h, cells were gently washed and mounted with ProLong Gold Antifade reagent with DAPI (Thermo Fisher Scientific) before visualization under an Eclipse TE2000-E fluorescence microscope (Nikon, Tokyo, Japan).

### Electron Microscopy

Cells were pelleted, fixed in 4% glutaraldehyde overnight, and post-fixed in osmium tetroxide. Cell pellet was dehydrated with an ascending series of ethanol and embedded in the epon mixture prepared with Agar 100, dodecyl succinic anhydride, methyl nadic anhydride, and tri-dimethylaminomethyl phenol. Samples were sectioned using an ultramicrotome and stained with uranyl acetate and lead citrate before observation with transmission electron microscope LEO LIBRA120 (Carl Zeiss, Oberkochen, Germany).

### Macrophage Intracellular Killing Assay

Culture medium of *H. pylori*-infected cells at 24 h post-infection (h.p.i.) was replaced with DMEM with 100 μg/ml of gentamycin for extracellular bacteria killing. After 1-h incubation, cells were washed and lysed with 0.1% saponin at 37°C for 15 min. Viable intracellular bacteria count was determined by calculating colony-forming units (CFU) of the serially diluted bacteria that were plated and cultured on chocolate agar plate.

### Real-Time Cell Analysis Assay and Transwell Migration Assay

Real-time cell migration was monitored using electronically integrated Boyden chamber CIM-16 plate in a xCELLigence RTCA DP instrument (ACEA Bioscience, San Diego, CA). Briefly, the lower chamber was detached and loaded with DMEM with 20% FBS, 100 ng/ml CCL2, and 1 µg/ml LPS from *E. coli* (O111:B4, Sigma Aldrich, USA). The upper chamber was reassembled and 50 µl of serum-free DMEM was added. CIM-16 plate was placed on RTCA instrument in a 37°C incubator for 1 h and background signal was measured for 5 min. Then, cells were seeded at 5 × 10^5^ cells/ml in DMEM media supplemented with 10% FBS in the upper chamber and allowed to equilibrate and attached at room temperature for 30 min before initiating measurement. Data were analyzed in a xCELLigence RTCA Software Pro V2.0 (ACEA Bioscience).

Conventional migration assay was performed using 8-μm 24-well transwell inserts. Cells were seeded in the upper chamber at 3 × 10^5^ cells/ml. Lower chamber was filled with DMEM with 20% FBS, 100 ng/ml CCL2, and 1 µg/ml LPS. After 24 h, non-migrated cells inside the insert were removed using a cotton tip while migrated cells were fixed with methanol for 10 min, stained with crystal violet staining, and visualized under a light microscope. Ten images from different fields were captured randomly for cell calculation. For migration assay using *H. pylori*, bacteria suspension was adjusted to 10^8^ cells/ml and heat-inactivated at 56°C for 30 min. Subsequently, inactivated bacteria were added at MOI 50:1 in the lower chamber and incubated for 24 h.

### Wound Healing Assay

Cells were seeded onto a 96-well plate at a concentration of 5 × 10^5^ cells/ml to achieve around 90% confluency. Media was changed into fresh DMEM supplemented with 10% FBS and a 10-µl pipette tip was used to scratch a wound in the middle of the well. Wound healing was recorded every 5 min with JuLi BRD04 live cell imager (NanoEntek, Pleasanton, CA) for 24 h. Area of wound was measured every 4 h using ImageJ (version 1.50b). Percentage of wound healed was calculated with the following equation, where T_0_ indicates 0 h; T_n_ indicates selected time point.


$$\text{Wound Healed }(\%)=\frac{\text{Wound siz}{\text{e}}_{\text{T}0}-\text{Wound siz}{\text{e}}_{\text{Tn}}}{\text{Wound siz}{\text{e}}_{\text{T}0}}\times 100\%$$

### Immunofluorescence Microscopy

Cells were seeded at 2 × 10^5^ cells/ml in a four-well chamber slide and incubated overnight. After 24-h treatment with 100 ng/ml CCL2 and 10 ng/ml LPS, cells were fixed with ice-cold methanol for 10 min, washed with PBS/0.01% Triton-X, and blocked with PBS/3% BSA. Cells were incubated with rabbit anti-mouse-β-actin antibody (Cell Signaling Technology, Beverly, MA) for 1 h followed by incubation with secondary antibodies Alexa Fluor 555-conjugated anti-rabbit IgG (H+L) F(ab’)_2_ (Cell Signaling Technology). Cells were washed with PBS/0.01% Triton-X again before counterstained with Prolong gold antifade DAPI reagent. Slide was viewed in a Leica TCS SP5 II confocal microscope (Leica Microsystems, Mannheim, Germany).

### Cell Proliferation Assay

Cells were seeded at 2 × 10^5^ cells/ml in different wells of four 96-well plate and incubated for 4 days. Each day, a plate was removed and processed. A total of 50 µl of 3-(4,5-dimethylthiazol-2-yl)-2,5-diphenyltetrazolium bromide (MTT) substrate was added and incubated for 1 h. Cell supernatant was removed carefully and replaced with 100 µl of DMSO. Absorbance reading at 570 nm was measured using a Synergy HTX Multi-Mode microplate reader (BioTek Instruments, Winooski, VT).

### NanoString Gene Expression Analysis

A total of 100 ng of RNA (50 ng/µl) was added to hybridization buffer, reporter, and capture probe sets (Cat: NNS_NAA-AKIT-012, Lot: CP6643X1), and hybridized at 65°C for 20 h. Subsequent sample preparation and scanning were performed using an automated nCounter Prep station for hybridization onto the sample cartridge (Cat: NNS_XT-CSO-MPATH1-1, Lot: RC7091X1) followed by scanning with Digital analyzer (NanoString Technologies, Seattle, WA). Data were analyzed using the nSolver Analysis Software V4.0. Two-step data normalization was performed using an internally developed Pipeline Pilot Tool (NAPPA) to account for the background using the spike in positive and negative controls, followed by input correction using housekeeping genes. Genes that had a mean value fewer than 10 counts across all samples were removed from analysis. NanoString gene expression data have been deposited to the National Center for Biotechnology Information (NCBI) Gene Expression Omnibus (GEO) repository available at https://www.ncbi.nlm.nih.gov/geo/query/acc.cgi?acc=GSE151269.

### ELISA

Enzyme-linked immunosorbent assay (ELISA) was carried out using ELISA MAX™ Deluxe Set Mouse (Biolegend) according to the manufacturer’s protocol. Cell lysate was used for pro-IL-1β detection since RAW264.7 cell lacks apoptosis-associated speck-like protein containing a Card (Asc), an inflammasome adaptor indispensable for IL-1β maturation (39). Supernatant from cell culture was used for the detection of TNFα.

### Statistics

Data were shown as mean ± standard deviation (SD). Data were analyzed with unpaired two-tailed Student’s *t*-test when comparing between two groups, in GraphPad Prism (GraphPad software, La Jolla, CA, www.graphpad.com). Statistical significance among three groups were analyzed using two-way ANOVA test. *p*-values of <0.05 were considered as statistically significant (* *p* < 0.05, ** *p* < 0.01, *** *p* < 0.001, **** *p* < 0.0001). Statistical analysis for NanoString data was performed by R-based statistic program by either loglinear regression or simplified negative binomial model and adjusted with the Benjamini–Yekutieli method in nCounter Advanced Analysis 2.0 (NanoString Technologies). Samples were considered significant if *p* < 0.01 (−log_10_ > 2).

## Results

### *H. pylori* Infection Upregulates Pdpn Expression in Macrophages

**Figure 1:**
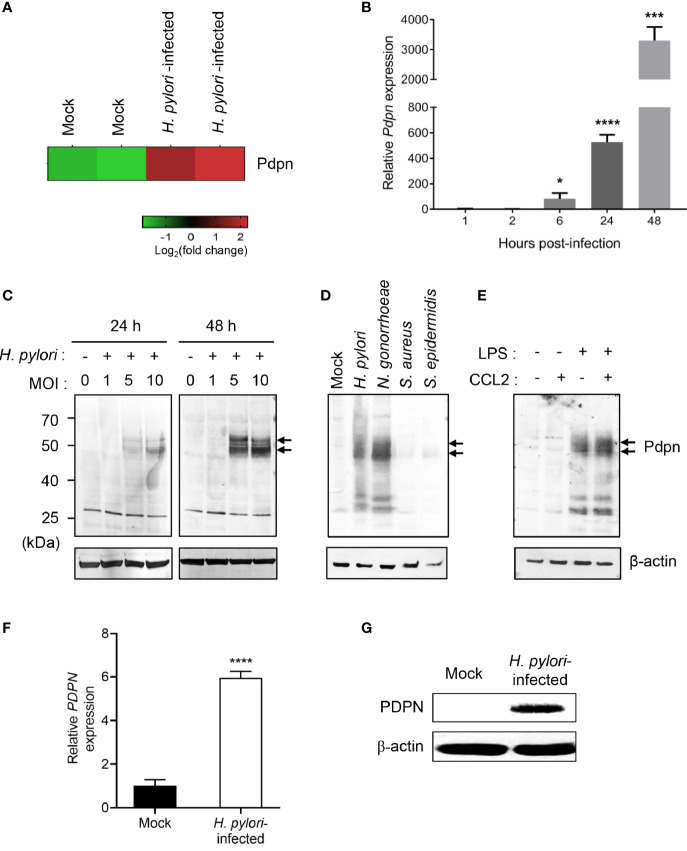
Upregulation of Pdpn expression in *H. pylori*-infected macrophages. **(A)** Microarray heatmap shows *Pdpn* gene expression in uninfected (mock) or *H. pylori* (MOI 10, 24 h)-infected RAW264.7 cells. Data were ran with two biological duplicates and were representative of two different probes. **(B)** Temporal *Pdpn* mRNA transcript expression of mock or *H. pylori* (MOI 10)-infected RAW264.7 cells. *y-*axis shows relative expression of *Pdpn* to *β*-*actin* internal control. Data were shown as mean ± *SD*, from two experiments ran in triplicate. Statistical significance was analyzed with unpaired Student’s *t*-test (*p < 0.05, ***p < 0.001, ****p < 0.0001) in comparison to level of uninfected control. **(C–E)** Immunoblot analyses show Pdpn protein expression in RAW264.7 cells under different stimulation conditions. Pdpn expressions were examined in **(C)** mock or *H. pylori* (MOI 1, 5, or 10)-infected cells at 24 or 48 h.p.i.; **(D)** mock, *H. pylori*, *N. gonorrhoeae*, *S. aureus*, and *S. epidermidis* (MOI 10, 24 h)-infected cells; and **(E)** LPS (1 ng/ml)-, CCL2 (100 ng/ml)-, or LPS plus CCL2-stimulated cells. Membranes were probed using anti-Pdpn (upper panel, as indicated by arrows) or anti-β-actin (lower panel) antibodies. Ladder indicates molecular weight, kDa: kiloDalton. Data were representative of at least two independent experiments. **(F, G)** THP-1 cells were uninfected (mock) or infected with *H. pylori* J99 strain at MOI 10 for 16 h. **(F)** *PDPN* mRNA transcript expression was examined by qRT-PCR method. *y-*axis shows relative expression of *PDPN* to *β*-*actin* internal control. Data are shown as mean ± *SD*, from two experiments run in triplicate. Student’s *t*-test was used for statistical analysis; ****p < 0.0001. **(G)** Immunoblot analysis shows PDPN protein expression in THP-1 cells in mock or *H. pylori* (MOI 10 for 16 h)-infected cells. Membranes were probed using anti-PDPN or anti-β-actin antibodies. Data were representative of at least two independent experiments.

Our previous microarray analysis revealed an altered transcriptional profile of multiple genes in *H. pylori*-infected macrophages (37). Out of the genes attained, we selected *Pdpn* gene for further investigation given its uncharacterized function in macrophage and intense upregulation following *H. pylori* infection (**Figure 1A**). To validate the microarray data, we infected RAW264.7 macrophages with *H. pylori* SS1 for examination at both transcriptional and translational levels. *H. pylori* SS1 was used in the current study as this strain is the mouse adapted strain widely employed in studies of *H. pylori* using mouse model (40). Quantitative RT-PCR results demonstrated a markedly elevated amount of *Pdpn* mRNA transcript in the *H. pylori*-infected cells relative to uninfected mock control (**Figure 1B**). *Pdpn* mRNA transcript was detected at 6 h.p.i. at approximately 83-fold and steadily increased up to 527-fold and 3,307-fold, at 24 and 48 h.p.i., respectively. At the protein level, Pdpn band was not detected in the uninfected cells but was apparent upon *H. pylori* infection at MOI ≥ 5, and increased in a time-dependent fashion (**Figure 1C**, arrows). We then hypothesized that the upregulation of Pdpn in response to *H. pylori* infection could also occur with other bacteria. Two gram-positive (*S. aureus* and *S. epidermidis*) and two gram-negative (*H. pylori* and *N. gonorrhoeae*) bacteria were selected for the test. Interestingly, we noted that Pdpn expression was strongly induced following infection with gram-negative but not gram-positive bacteria (**Figure 1D**). Induction of Pdpn was likely triggered by bacteria surface lipopolysaccharide (LPS), as shown in **Figure 1E** and a previous report (24). Additional stimulation with C-C chemokine ligand 2 (CCL2) chemoattractant showed no additive or synergistic effect on Pdpn expression (**Figure 1E**). Further confirmation of PDPN expression in human cells was also carried out in human THP-1 monocytic macrophages using *H. pylori* J99 strain, a highly pathogenic strain in human, where upregulation of expression was observed at both mRNA and protein levels (**Figures 1F, G**).

### Pdpn Deletion Does Not Affect Macrophage Phagocytic or Bactericidal Activities

To further investigate the functional role of Pdpn in macrophages, we disrupted *Pdpn* gene in the parental RAW264.7 cells using CRISPR/Cas9 gene editing technology. Two stable clones with early non-sense mutation were selected (**Supplementary Figures S1A, B**). Three-dimensional protein structure prediction suggested that the mutations resulted in disrupted alpha helix folding at the N terminal extracellular domain of Pdpn protein, indicating loss of protein function (**Supplementary Figure S1C**). The first clone exhibited a partial (~70%) reduction in expression with a lower molecular weight indicative of protein truncation and was labeled as podoplanin knockdown with truncation (*Pdpn-*KD/TC), whereas the second clone showed no detectable protein and thus was labeled as podoplanin knockout (*Pdpn-*KO) (**Supplementary Figure S1D**). Most of the following analyses were conducted using *Pdpn-*KO cells.

A previous study reported high phagocytic activity of podoplanin-expressing inflammatory macrophages (24). Hence, we first assessed the ability of *Pdpn-*KO cells in phagocytosing *H. pylori*. In transmission electron microscopy images, internalized bacteria in *Pdpn-*KO cells can be clearly detected as densely stained, curved rod structure in the phagocytic vacuoles (**Supplementary Figure S2A**). By using FITC-labeled *H. pylori*, immunofluorescence images revealed that *Pdpn-*KO cells effectively engulfed bacteria comparable to the wild-type parental cells (**Supplementary Figure S2B**). Flow cytometric analysis at 1 h.p.i. suggested an effective albeit minimal delay in the phagocytic ability of *Pdpn-*KO; data at 4 h.p.i. demonstrated that >98% of *Pdpn-*KO successfully phagocytosed the bacteria (**Supplementary Figure S2C**). In intracellular bactericidal assay, no significant difference was observed in the CFU of the viable *H. pylori* that remained within the *Pdpn-*KO and wild-type macrophages following infection (**Supplementary Figure S2D**), suggesting that *Pdpn* **-**deletion caused no direct defect in bacterial uptake and killing.

### Pdpn-Knockout Cells Exhibit Impaired Migration Activity

**Figure 2:**
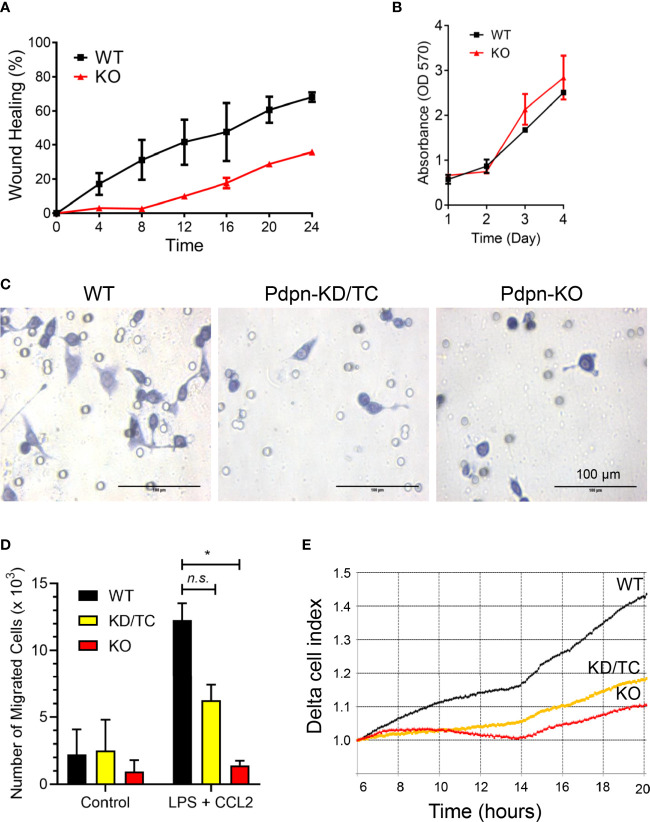
Impaired motility of LPS-stimulated macrophages following *Pdpn*-deletion. **(A)** Graph shows percentages of wound healing in WT and *Pdpn*-KO cells. Confluent cells were scratched and viewed under NanoEntek JuLi BRD04 live cell imager for 24 h. Area of wound was measured every 4 h using ImageJ software. Note that *Pdpn*-KO demonstrated impaired wound healing ability as compared to WT cells. Data were presented as mean ± *SD* and were representative of three independent experiments. See **Supplemental Video 1**. **(B)** Comparison of cell proliferation rate among the non-stimulated wild type (WT) and *Pdpn*-knockout (KO) stable clones using MTT assay. Similar number of cells were seeded and cultured over a period of 4 days. Cell growth was measured each day by adding MTT solution for 4 h, replaced with DMSO, before measuring on a plate reader. All samples were run in triplicate. Data are presented as mean ± *SD* from duplicate readings and are representative of two independent experiments. **(C)** Representative images of migrated cells for wild-type, *Pdpn*-KD/TC, and *Pdpn*-KO cells in transwell migration assay. Cells seeded at upper chamber of 8-μm transwell insert were induced with 20% FBS, 100 ng/ml CCL2, and 1 µg/ml LPS from the lower chamber and processed with crystal violet staining after 24 h. Circles indicate the pores in the transwell. Scale bars indicate 100 μm. **(D)** Histogram shows number of migrated cells in transwell migration assay. Data were shown as mean ± *SD* from duplicates and were representative of three independent experiments. Statistical significance was analyzed with two-way ANOVA test (*F* value: 5.77; degree of freedom: 6; *p < 0.05; *n.s*., not significant). **(E)** Real-time migration rate of wild-type, *Pdpn*-KD/TC, and *Pdpn*-KO cells in RTCA analysis. Cells seeded at the upper chamber of CIM-16 plate were induced with 20% FBS, 100 ng/ml CCL2, and 1 µg/ml LPS, from the lower chamber. Data were normalized at the sixth hour time point and are representative of two independent experiments.

Given that fewer CD11b^+^ F4/80^+^ cells are recruited to the site of inflamed peritonitis in *Vav*-Cre *Pdpn*-floxed mice (30), we postulated that Pdpn mediates macrophage migration. Using wound healing assay, we observed that *Pdpn-*KO cells were inefficient in closing the scratched area compared to wild-type cells (**Figure 2A**). We exclude the possibility that the inefficient wound healing ability was a result of defective cell proliferation capability, as shown by parallel cellular proliferation rates over a period of 4 days between wild-type and knockout cells (**Figure 2B**). Strikingly, live recording demonstrated static or limited movement of *Pdpn-*KO cells compared to highly motile wild-type cells (**Supplementary Video S1**).

We further compared cellular migration of parental and *Pdpn-*KO cells towards LPS and CCL2 chemoattractant. The number of the migrated cells in the Boyden chamber assay was remarkably reduced by approximately 50% and >90%, in *Pdpn-*KD/TC and *Pdpn-*KO cells, respectively, compared to wild-type control (**Figures 2C, D**). Notably, the morphology of the mutant cells appeared smaller and rounder compared to that of the migrated parental cells (**Figure 2C**). Next, we repeated the migration assay by using *H. pylori* induction. The heat-inactivated form of *H. pylori* was used to avoid the migrated cell count from being affected by live bacterial infection-mediated cell death. We noted that wild-type cells migrated efficiently towards lower chamber containing the heat-inactivated form of *H. pylori*. Consistent with the above induction using LPS and CCL2, a significantly lower number of migrated *Pdpn-*KD/TC and *Pdpn-*KO cells were induced by *H. pylori*, suggesting the defective migratory activity (**Supplementary Figure S3**). Results from an impedance-based real-time cell analyzer analysis demonstrated that the cellular migration rate towards CCL2 chemoattractant increased steadily over time in the wild-type parental cells, but it was substantially declined in *Pdpn-*KO or *Pdpn-*KD/TC cells (**Figure 2E**). These observations collectively suggest that Pdpn modulates cell migration of activated macrophages.

### Filopodia Formation Is Diminished by Pdpn Deletion and Intensified by Pdpn Overexpression

**Figure 3:**
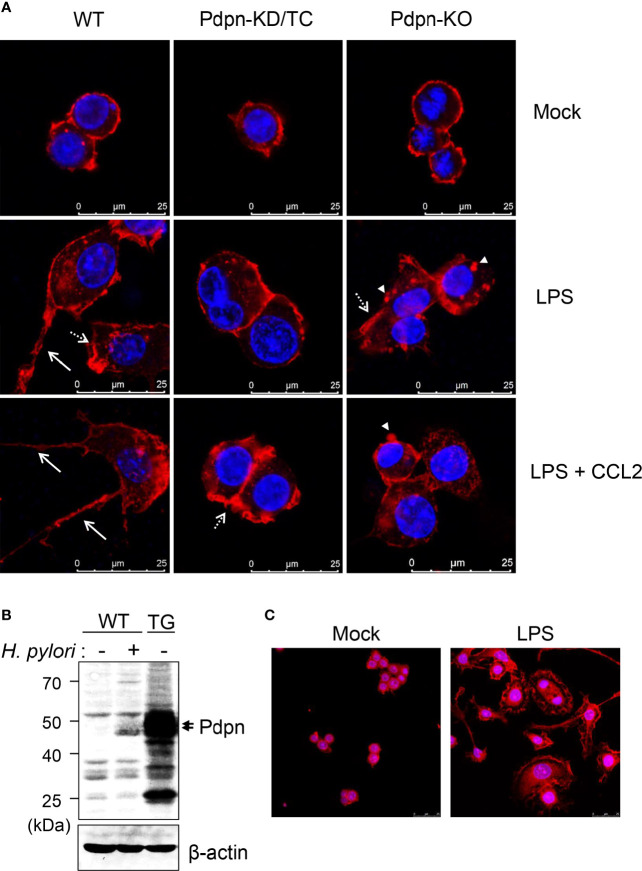
Altered filopodia formation in the *Pdpn*-knockout and overexpressed cells. **(A)** Representative immunofluorescence images of wild-type, *Pdpn*-KD/TC, and *Pdpn*-KO cells with or without treatment with 10 µg/ml LPS (and 100 ng/ml CCL2). Note that actin cytoplasmic tails or filopodia protrusions (arrow) were only detected in the wild type, while membrane ruffles or lamellipodia (broken arrow) and podosomes (arrowhead) can be observed in the mutant clones. Cells were stained with anti-β-actin antibody followed by Alexa Fluor 555-conjugated rabbit anti-rabbit IgG (H+L) F(ab’)_2_ to view actin cytoskeleton (red) and nucleus were counterstained with DAPI (blue). Scale bars represent 25 μm. **(B)** Generation of *Pdpn*-overexpression clone. Immunoblot analysis of Pdpn in uninfected or *H. pylori* (MOI 10, 24 h)-infected wild-type cells, and unstimulated *Pdpn*-TG stable clone. Membranes were probed using anti-Pdpn or anti-β-actin antibodies. Ladder indicates molecular weight, kDa: kiloDalton. **(C)** Representative immunofluorescence images of *Pdpn*-TG cells with or without treatment with 1 µg/ml LPS. Note the extensive filopodia protrusions around the activated cells. Cells were stained as described in **(A)**. Scale bars represent 25 μm.

When cells were stained and visualized under confocal microscopy, there was no overt differences observed in the WT, *Pdpn-*KD/TC, and *Pdpn-*KO cells prior to activation (**Figure 3A**, upper panel). Upon treatment with LPS (and CCL2), wild-type cells displayed increased cellular size with extended filopodia protrusion and long cytoplasmic tail formation, suggesting active actin polymerization and directional cell migration. In contrast, LPS (and CCL2) treatment failed to induce the formation of filopodia and long cytoplasmic tail in the mutant clones (**Figure 3A**, as indicated by arrows, medium, and lower panels). Although *Pdpn-*KD/TC and *Pdpn-KO* cells lacked long filopodia, the formation of lamellipodia and podosome structures (**Figure 3A**, as indicated by broken arrow and arrowhead) can still be detected.

To further confirm the involvement of Pdpn in filopodia formation, a stable clone ectopically expressing *Pdpn* was generated using a retrovirus transduction system. The level of Pdpn expression in the *Pdpn-*TG cells was strongly evident at the protein level under unstimulated condition, at a greater level compared to that of *H. pylori*-stimulated WT cells (**Figure 3B**). Intriguingly, an explosive amount of filopodia protrusion was detected in the *Pdpn-*TG cells after LPS stimulation (**Figure 3C**). Absence of filopodia in the knockout cells and its excessive presence in the transgenic cells underscore an essential role of *Pdpn* in controlling filopodia formation. It should be noted that this phenomenon was not observed in unstimulated Pdpn-TG cells, suggesting that additional factors derived from cell signaling are indispensable for initiating actin polymerization process compulsory for filopodia formation.

### Pdpn Deletion Abrogates the Production of Interleukin-1β From the Activated Macrophages

**Figure 4:**
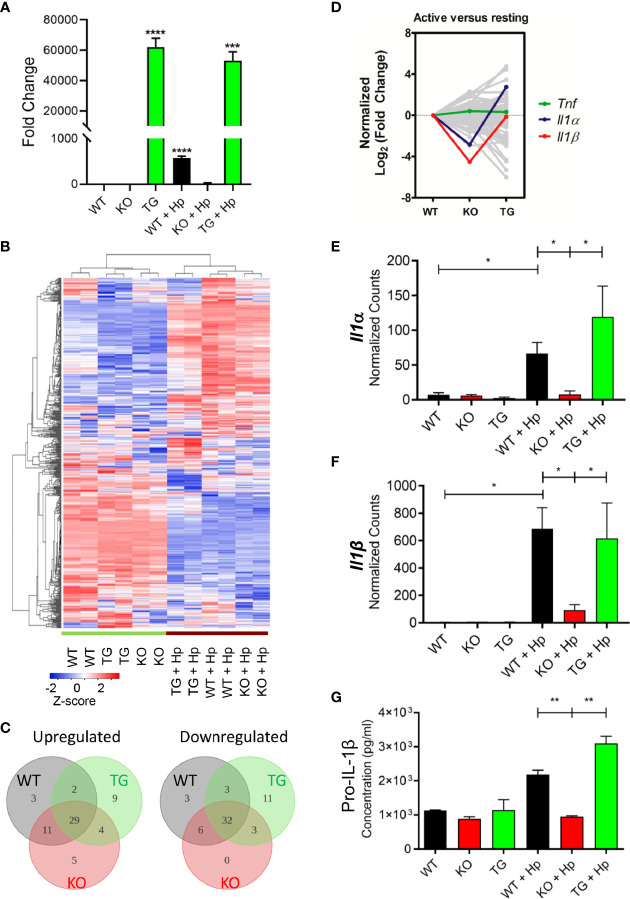
*Pdpn*-deletion inhibits the production of IL-1β proinflammatory cytokine. **(A)** Bar chart depicting the fold changes of *Pdpn* mRNA transcript expression in the wild type (WT), *Pdpn*-knockout (KO), and *Pdpn*-transgenic (TG) cells, before and after *H. pylori* infection at MOI 10 for 24 h (as indicated by +Hp). The mRNA transcript was isolated from each cell, reverse transcribed to cDNA and subjected to quantitative RT-PCR analysis. *y-*axis shows normalized fold change of *Pdpn* over *β*-*actin* internal control. Data were shown as mean ± *SD* of triplicates obtained from representative of two independent experiments. Statistical significance was analyzed with unpaired Student’s *t*-test (***p < 0.001, ****p < 0.0001). **(B)** Heatmap showing the gene expression and hierarchical cluster analysis of NanoString data. Color intensity reflects the *Z*-scores of the RNA abundance. Red represents upregulation while blue represents downregulation. All samples were run as biological duplicates. Statistical analysis was performed by R-based statistic program by either loglinear regression or simplified negative binomial model and adjusted with the Benjamini–Yekutieli method in nCounter Advanced Analysis 2.0 software. **(C)** Venn diagrams show the number of differentially upregulated (log_2_ fold change > 2, left panel) or downregulated (log_2_ fold change < -2, right panel) genes following *H. pylori* infection. Subsets are WT (gray), KO (red), and TG (green). Overlap region indicated the number of common differentially expressed genes among comparison groups. Diagram was generated by Lucidchart platform. **(D)** Line chart showing the normalized trend of differentially expressed genes following infection. *y-*axis represents the normalized log_2_ fold change of each gene in WT, KO, or TG samples over the log_2_ fold change of WT. Sharp “V” trendline was observed for *Il1α* and *Il1β* in KO cells, indicating drastically lower increment following infection when compared to both WT and TG cells. Differential expressions were computed in the nSolver Advance Analysis Software 2.0. **(E, F)** Bar charts show normalized RNA transcript counts of **(E)** *Il1α* and **(F)** *l1β* from NanoString analysis. Data are shown as mean ± *SD* of duplicate samples. Statistical significance was analyzed with unpaired Student’s *t*-test (*p ≤ 0.05). **(G)** Bar chart shows ELISA analysis of pro-IL-1β production in cell lysate. *y-*axis represents cytokine concentrations in pg/ml. Data were shown as mean ± *SD* of duplicates obtained from representative of two independent experiments. Statistical significance by unpaired Student’s *t*-test (**p < 0.01).

To further investigate the changes of *Pdpn-*KO and *Pdpn-*TG cells at the molecular level, mRNA transcripts were analyzed using a NanoString nCounter^®^ PanCancer Pathways panel that included genes in immune-oncology, innate, and adaptive immune responses. Prior to the assay, the expression levels of *Pdpn* among the parental WT, *Pdpn-*KO, and *Pdpn-*TG cells were verified before and 24 h after *H. pylori* infection using RT*-*PCR (**Figure 4A**). For WT cells, *Pdpn* expression was low in resting cells, and increased at approximately 600-fold after *H. pylori* infection. As expected, no *Pdpn* expression was observed in KO cells, while strong expression of >50,000-fold was detected in *Pdpn-*TG cells regardless of infection status.

In general, wild-type, *Pdpn-*KO, and *Pdpn-*TG cells displayed a highly homogeneous gene expression profile that altered comparably upon *H. pylori* infection, as depicted by the heatmap (**Figure 4B**), suggesting minimal influence of *Pdpn* disruption on the transcriptional programming of the macrophages. Among the top 10 upregulated genes following *H. pylori* infection in wild-type cells were cytokines for myeloid differentiation *Colony stimulating factor 2* and *3* (*Csf2, Csf3*), proinflammatory cytokines *interleukin*-*1β* (*Il1β*) and *Il6*, *chemokine C*-*X*-*C ligand 2* (*Cxcl2*), as well as signaling molecules *Suppressor of cytokine signaling 3* (*Socs3*) and *Linker of T cell activation* (*Lat*) (**Supplementary Figure S4A**, red dot cluster), indicating a robust immune response in the macrophages following *H. pylori* infection. A similar pattern is observed in KO and TG groups following infection (**Supplementary Figures S4B, C**). Venn diagrams also depict most of the differentially expressed genes (log_2_ fold change > 2 or <−2) following infection for KO and TG groups, which were in common to that of WT (**Figure 4C**).

Despite similar expression changes across all cells following infection, we noted that *Il1β* was missing from the list of top 10 upregulated genes of the activated *Pdpn*-KO cells (**Supplementary Figure S4B**, red dot cluster), but retained in *Pdpn-*TG cells (**Supplementary Figure S4C**, red dot cluster). Normalized log_2_ fold changes highlighted expression of *Il1β* and *Il1α* in sharp “V” trendlines, reflecting lower expressions in activated *Pdpn*-KO compared to both WT and *Pdpn*-TG cells (**Figure 4D**, red and blue lines). Other genes, for example, *Tumor necrosis factor* (*Tnf*) exhibited rather equivalent level of fold changes across three comparison groups (**Figure 4D**, green line).

The mRNA transcripts of *Il1β* were undetectable (<10 counts) in resting macrophages but were drastically increased to 684.8 ± 110.7 and 616.6 ± 182.3 counts in the activated wild-type and *Pdpn-*TG cells, respectively (**Figure 4E**). However, in *Pdpn-*KO cells, *H. pylori*-mediated expression was merely 93.4 ± 27.8 counts, approximately sevenfold lower than that of wild-type and *Pdpn*-TG cells. Similarly, transcription of *Il1α* was greatly augmented to 66.5 ± 11.0 and 119.1 ± 11.4 counts in wild-type and *Pdpn-*TG cells post-infection (**Figure 4F**), but not in *Pdpn-*KO cells. In contrast, no drastic changes were observed in the transcript levels of tumor necrosis factor (*Tnf*) across all cells post-infection (**Supplementary Figure S5A**). Production of pro-IL-1β and TNFα was verified by using ELISA (**Figure 4G**, **Supplementary Figure S5B**). Concentration of pro-IL-1β in cell lysate increased at two- to threefold in the activated WT and *Pdpn-*TG cells, while no such increase was detected in *Pdpn-*KO cells, in accordance with the mRNA transcript level (**Figure 4G**).

### Pdpn Regulates the Transcriptional Expression of Filamin C Actin-Binding Protein

**Figure 5:**
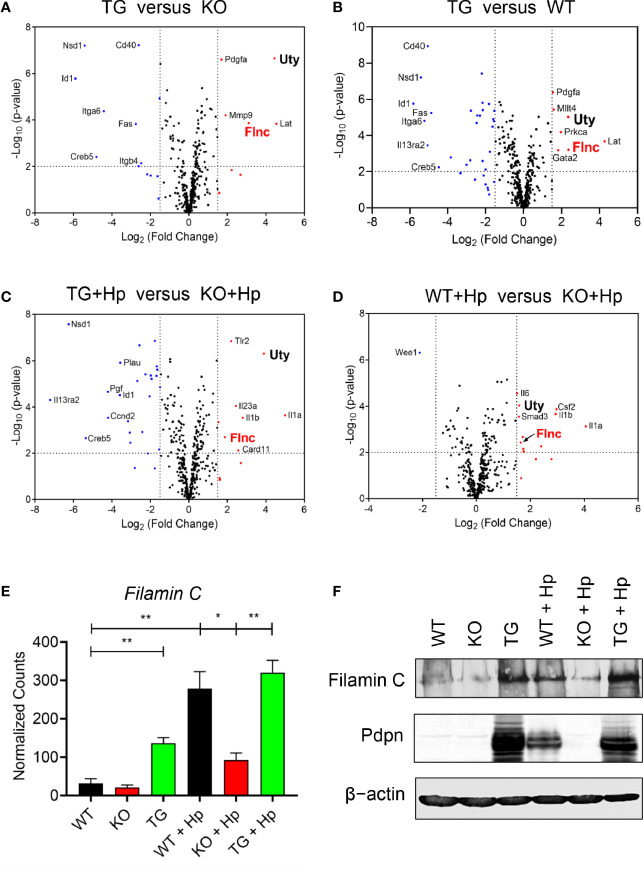
*Pdpn* regulates the expression of Filamin C. **(A–D)** Volcano plots showing differential gene expression in groups of wild type (WT), *Pdpn*-knockout (KO) and *Pdpn*-transgenic (TG) cells before and after *H. pylori* infection at MOI 10 for 24 h (as indicated by +Hp). Comparison was made between the group with a high *Pdpn* expression *versus* the group without or with a lower *Pdpn* expression, as follows: **(A)** *Pdpn*-TG *versus Pdpn*-KO; **(B)** *Pdpn*-TG *versus* WT; **(C)** Activated *Pdpn*-TG *versus* activated KO; and **(D)** Activated WT *versus* activated *Pdpn*-KO. Note that a gene *Filamin C* (*FlnC*) was repetitively shown in all four groups of comparison, indicating its consistent upregulation by high *Pdpn* expression. Vertically broken lines demarcate log_2_ (fold change) at >1.5 or <−1.5. Horizontally broken lines demarcate statistical significance (−log_10_ *p*-value > 2, or *p* < 0.01) by R-based program in nSolver Advanced Analysis 2.0 software. Significantly upregulated and downregulated genes were highlighted in red and blue dots, respectively. **(E)** Bar charts show normalized RNA transcript counts of *Filamin C* from NanoString analysis. Data are shown as mean ± *SD* of biological duplicate samples. Statistical significance by unpaired Student’s *t*-test (*p ≤ 0.05; **p ≤ 0.01). **(F)** Immunoblot analysis of Filamin C Cell lysate from the uninfected or *H. pylori* (MOI 10, 24 h)-infected WT, *Pdpn*-KO, and *Pdpn*-TG cells were examined. Membranes were probed with antibodies to Filamin C, Pdpn, and β-actin as loading control. Data shown are representative data of two independent experiments.

We then compared, in various pairwise combinations, the transcriptional outputs of knockout, transgenic, and wild-type cells in uninfected and *H. pylori*-infected contexts (**Figures 5A–D**). We were intrigued by the expression of a particular gene, *Filamin C* (*Flnc*), which was consistently greater in the high *Pdpn-*expressing cells compared to low/no *Pdpn-*expressing cells before (**Figures 5A, B**) or after *H. pylori* infection (**Figures 5C, D**). *Filamin C* encodes for an actin-binding protein essential for muscle movement (41) and is involved in actin cytoskeleton modulation (42). Given its actin regulation function, it is plausible that Filamin C is involved in macrophage migration *via* a Pdpn-dependent pathway. Comparison of normalized transcript counts showed a low expression of *Filamin C* in the absence of Pdpn (**Figure 5E**). Prior to infection, *Filamin C* was elevated by 4.3-fold in *Pdpn-*TG cells. *H. pylori* infection resulted in an 8.7-fold and 10-fold upregulation of *Filamin C* from 31.7 ± 8.3 in resting WT macrophages to 278.3 ± 31.0 counts and 320.1 ± 22.5 counts in activated WT and *Pdpn-*TG macrophages. However, *H. pylori* infection-mediated *Filamin C* gene upregulation was relatively low, at merely threefold increment to 92.3 ± 12.9 counts in *Pdpn-*KO cells. At the protein level, Filamin C protein was greatly decreased in *Pdpn-*KO and augmented in *Pdpn-*TG cells (**Figure 5F**), correlating with the RNA transcript data, suggesting dependency of Filamin C on the Pdpn signaling pathway.

*Ubiquitously transcribed tetratricopeptide repeat containing, Y-linked* (*Uty*) was also consistently greater in the high *Pdpn-*expressing cells compared to low/no *Pdpn-*expressing cells before (**Figures 5A, B**) or after *H. pylori* infection. *Uty* encodes for a histone demethylase enzyme although its significance in macrophage migration remains to be identified (43).

## Discussion

The present study reports that the *Pdpn* gene that encodes for a transmembrane molecule was significantly upregulated in macrophages ensuing *H. pylori* infections. In addition to *H. pylori*, stimulation by LPS or other gram*-*negative bacteria infection were found sufficient to trigger *Pdpn* expression in macrophages, as reported in the current (**Figure 1**) and previous studies (24). Pdpn can also be induced by TLR1/2 and TLR2/6 agonists in addition to LPS (24); however, it is still unclear if other *H. pylori* virulent factors such as CagA can trigger its expression. Using the *Pdpn-*knockout cell model generated in the present study, we discovered that phagocytosis and bactericidal activities were not perturbed. Although a previous study reports that a subset of *Pdpn-*expressing macrophages are highly phagocytic (26), our data using knockout cell model rectify that there is no direct role of *Pdpn* in phagocytosis activity. Rather, we anticipate that high phagocytic activity in *Pdpn-*expressing cells is a consequence of aggressive cell movement directed towards the bacteria, as cell migratory activity was significantly attenuated in *Pdpn-*disrupted cells. Furthermore, we also report impeded filopodia protrusion following *Pdpn* deletion and extensive branched filopodia when *Pdpn* was ectopically overexpressed. Despite diminished filopodia, *Pdpn-*deleted cells demonstrated lamellipodia formation. Filopodia extend beyond lamellipodia and control effective directional cell movement (44). Filopodia and lamellipodia utilize different modes of actin polymerization assembly. For instance, depletion of capping protein results in loss of lamellipodia but extensive filopodia protrusion, whereas Ena/VASP deficiency resulted in membrane ruffling without filopodia (45). Mechanistically, our findings highlight that the defective cellular migration in the absence of *Pdpn* is attributable to a consequence of altered *Filamin C* expression. We anticipate that Pdpn may promote *Filamin C* transactivation through activating RhoGTPase signaling pathway, but further investigation is required to confirm this notion (10). This study adds novel knowledge to the mechanism and function of Pdpn, on top of the previous reports that credited its role predominantly to its extracellular interaction with Clec2 (13).

As implicated in the migration and would healing assays, motility was significantly impaired in *Pdpn-*KO and *Pdpn-*KD/TC cells as a result of diminished filopodia elongation. Filopodia formation is important to control directional cell movement. The involvement of Pdpn in migration has been established using non-immune cells such as mouse embryonic fibroblasts and cancer cells (8, 46–48). Our findings extend its role in regulating migration of immune cells. Interestingly, a previous study that relates Pdpn to dendritic cells motility suggests a model that is close to cancer metastasis, in which binding of Clec2 on dendritic cells to Pdpn on the stroma network activates actin rearrangement and promotes movement of dendritic cell in the lymphatic system (17). Since *in vitro* knockout setting was utilized in the current study, we suggest that Pdpn regulating macrophage migration is independent of external interaction with Clec2. Rather, our findings highlight an association between Pdpn with Filamin C, a member of actin-crosslinking protein family that stabilizes and links actin web to the cell membrane (49). Patients with mutated *Filamin C* gene demonstrate high incidence of cardiomyopathy (50). Filamin C maintains the structural integrity of skeletal and cardiac muscles by interacting with the sarcomeric Z-disc (41, 51). Given that Pdpn is indispensable in heart development, we anticipate that Filamin C activity in cardiomyocytes is potentially dependent on Pdpn signaling, but further analysis is required to validate this notion. Notably, *H. pylori* infection promotes aggressive metastasis of gastric epithelial cells by downregulating Filamin C level (52). Filamin A, another family member of filamin, has been implicated in Rac1/Cdc42-mediated monocyte migration, but the role of Filamin C remains understudied (53). Our findings warrant further study to clarify its link with Pdpn during immune cell migration.

*H. pylori* is a class I carcinogen that causes gastric adenocarcinoma through prolonged colonization, triggering chronic inflammation and destruction of gastric epithelial layer, which can eventually lead to chronic gastritis and gastric adenocarcinoma in humans. The current study suggests a correlation of *Pdpn* expression with the degree of *H. pylori* infection-mediated inflammation, through regulation of IL-1β proinflammatory cytokine. Production of IL-1β was evidently impaired in *Pdpn-*KO while enhanced in *Pdpn-*TG cells, as outlined in **Figure 4**. Increased IL-1β production aggravates development of gastric cancer by repressing secretion of gastric acid and orchestrating inflammatory response (54). Hence, we anticipate that a Pdpn inhibitor could be applied to alleviate inflammation and cancer in *H. pylori*-infected patients. On the other hand, Pdpn-Clec2 axis between macrophages and platelets is responsible for the local inflammatory response in either the peritoneum or liver during *E. coli* infection (30, 55) as well as in acetaminophen induced acute liver injury (56). *Pdpn* is also expressed by primary microglial cells during inflammation in traumatic brain injury (57). Our results acquired through examination of *Pdpn*-knockout and *Pdpn*-transgenic models provide evidence of Pdpn in controlling inflammatory diseases as its presence is essential for *IL-1β* cytokine expression and secretion.

Collectively, our findings suggest that Pdpn is crucial in regulating actin filament reorganization to enhance cell motility and filopodia extension, by altering Filamin C protein level. This study is limited by the employment of an *in vitro* infection model that may not accurately reflect the true complexity of host*–*pathogen interaction in an *in vivo* infection scenario. Nevertheless, this study opens up avenues for further research into clarifying the downstream mechanisms by which Pdpn modulates macrophage biology and suggests that Pdpn blockade may provide a new approach to relieve *H. pylori*-associated chronic inflammation.

## Data Availability Statement

The datasets presented in this study can be found in online repositories. The names of the repository/repositories and accession number(s) can be found in the article/**Supplementary Material**.

## Author Contributions

CYL and WFW conceived and designed the study. YYC, GMYT, KCF, CYQL, and YTC performed and analyzed the experiment. YYC and GMYT wrote the paper while YYC, GMYT, KCF, CYQL, HCC, and WFW reviewed and edited the paper. JV contributed reagents and tools. WFW and SA acquired funding. All authors contributed to the article and approved the submitted version.

## Funding

This work was supported by Fundamental Research Grant Scheme FRGS/1/2019/SKK06/UM/02/4 (FP133-2019A) from the Malaysia Ministry of Higher Education to SA and a Young Investigator Fund (IF039-2017) from the Institut Mérieux, France to WFW. YYC is supported by a Doctoral Scholarship from the Malaysia Public Service Department (JPA).

## Conflict of Interest

The authors declare that the research was conducted in the absence of any commercial or financial relationships that could be construed as a potential conflict of interest.

## Publisher’s Note

All claims expressed in this article are solely those of the authors and do not necessarily represent those of their affiliated organizations, or those of the publisher, the editors and the reviewers. Any product that may be evaluated in this article, or claim that may be made by its manufacturer, is not guaranteed or endorsed by the publisher.
