# Corrigendum: Podoplanin Drives Motility of Active Macrophage *via* Regulating Filamin C During *Helicobacter pylori* Infection

**DOI:** 10.3389/fimmu.2022.857795

**Published:** 2022-02-02

**Authors:** Yi Ying Cheok, Grace Min Yi Tan, Keith Conrad Fernandez, Yee Teng Chan, Chalystha Yie Qin Lee, Heng Choon Cheong, Chung Yeng Looi, Jamuna Vadivelu, Suhailah Abdullah, Won Fen Wong

**Affiliations:** ^1^ Department of Medical Microbiology, Faculty of Medicine, University of Malaya, Kuala Lumpur, Malaysia; ^2^ School of Bioscience, Taylor’s University, Subang Jaya, Selangor, Malaysia; ^3^ Department of Medicine, Faculty of Medicine, University of Malaya, Kuala Lumpur, Malaysia

**Keywords:** podoplanin, macrophage, *Helicobacter pylori*, cell migration, Filamin C, interleukin-1β

There is an error in the Funding statement. The correct number for “Fundamental Research Grant Scheme from the Malaysia Ministry of Higher Education” is “FRGS/1/2019/SKK06/UM/02/4 (FP133-2019A)”.

The authors apologize for this error and state that this does not change the scientific conclusions of the article in any way. The original article has been updated.

## Publisher’s Note

All claims expressed in this article are solely those of the authors and do not necessarily represent those of their affiliated organizations, or those of the publisher, the editors and the reviewers. Any product that may be evaluated in this article, or claim that may be made by its manufacturer, is not guaranteed or endorsed by the publisher.

