# Supplementary material for: Podoplanin Drives Motility of Active Macrophage via Regulating Filamin C During Helicobacter pylori Infection
Source: Front Immunol. 2021 Oct 11;12:702156. doi: 10.3389/fimmu.2021.702156 (PMC8543000; doi:10.3389/fimmu.2021.702156)
Supplement: Supplementary file 1 [file DataSheet_1.docx]

Supplementary Material

Podoplanin drives motility of active macrophage via regulating Filamin C during *Helicobacter pylori* infection

Yi Ying Cheok^1^, Grace Min Yi Tan^1,2^, Keith Conrad Fernandez^1,3^, Yee Teng Chan^1^, Chalystha Yie Qin Lee^1,4^, Heng Choon Cheong^1^, Chung Yeng Looi^5^, Jamuna Vadivelu^1^, Suhailah Abdullah^6^, Won Fen Wong^1^*

^1^Department of Medical Microbiology, Faculty of Medicine, University of Malaya, Kuala Lumpur, WP 50603, Malaysia

^2^Present address: Department of Microbiology and Immunology, University of Otago, Dunedin 9016, New Zealand

^3^Present address: Immunology and Microbial Pathogenesis Program, Weill Cornell Graduate School of Medical Sciences, Cornell University, New York, NY 10065, USA

^4^Present address: School of Health and Biomedical Sciences, RMIT University, Bundoora, VIC 3083, Australia

^5^School of Bioscience, Taylor’s University, Subang Jaya, Selangor 47500, Malaysia

^6^Department of Medicine, Faculty of Medicine, University of Malaya, Kuala Lumpur, WP 50603, Malaysia.

*** Correspondence:**Dr. Wong Won Fen
wonfen@um.edu.my

# Supplementary Figures

**
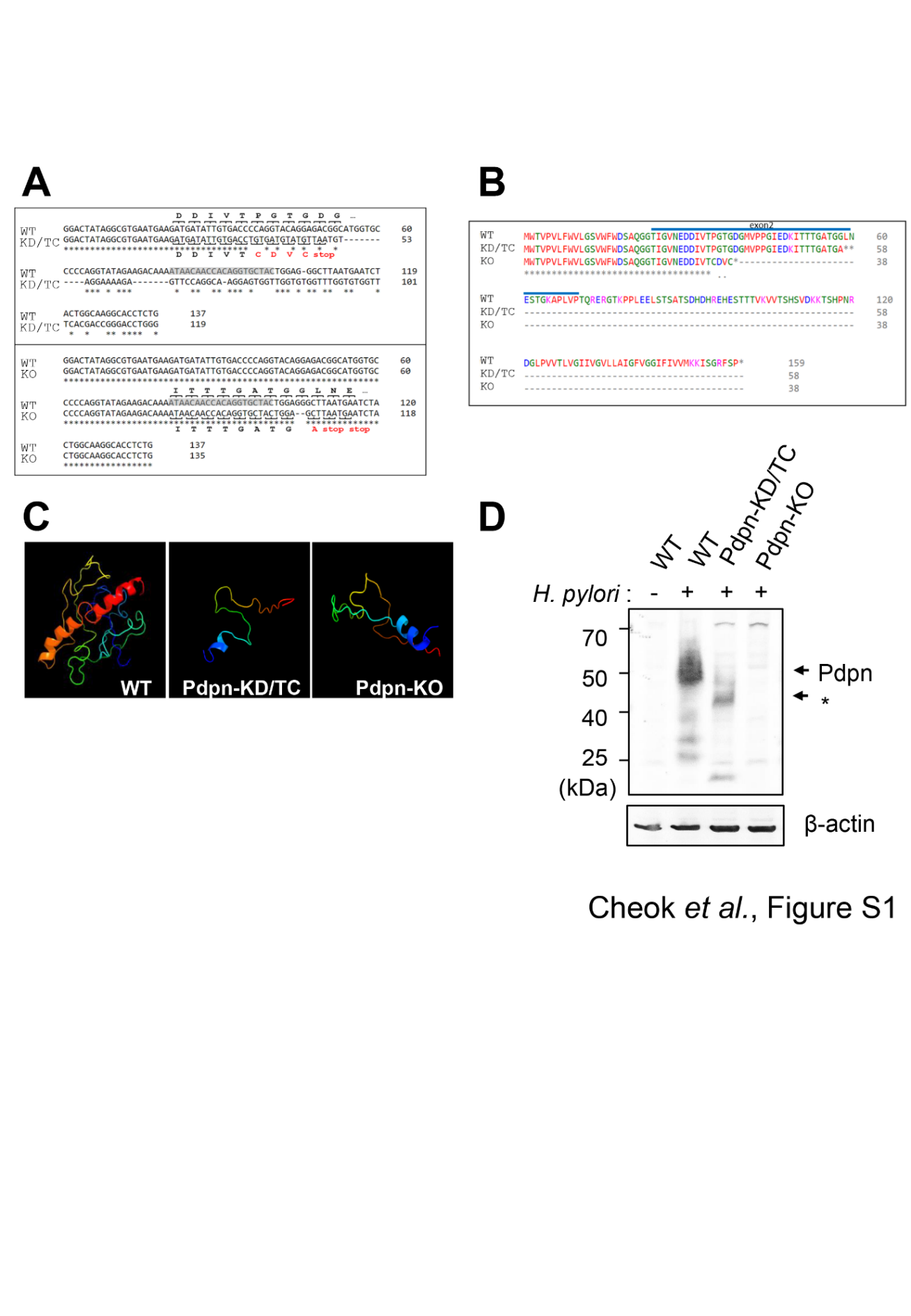
Supplementary Figure S1. Generation of Pdpn knockout cells via genome editing.**

**(A)** Sequencing alignment of the *Pdpn* exon 2 from wildtype (WT), *Pdpn*–KD/TC and *Pdpn*–KO mutant clones that were generated through CRISPR/Cas9 gene editing method by targeting a selected fragment (shaded region) in *Pdpn* exon 2. *Pdpn*–KD/TC was mutated from nucleotide 36 of exon 2 onwards, resulting in missense mutations in four amino acids followed by a TAA stop codon; while for *Pdpn*–KO, deletion of two guanines at positions 106 and 107 of exon 2 adjacent to the CRISPR target site resulted in a frameshift mutation that generated two tandem missense stop codons (TAA TGA) after the mutated site. Asterisks (*) show gene similarity between the WT and mutant clones. Amino acids and stop codons encoded by nucleotides adjacent to the mutated sites are as highlighted in red. **(B)** Multiple alignment of the Pdpn protein from parental wildtype (WT) RAW264.7 cells, Pdpn–knockdown/truncated (KD/TC) and Pdpn–knockout (KO) stable clones. The genetic changes caused an early protein truncation beginning at the 39th and 59th amino acid (aa) residues in respective mutant clones. Blue line indicates the amino acids encoded by the exon 2 region. Asterisks (*) represent similarity between the WT and mutant clones. **(C)** Three-dimensional protein structure prediction of wildtype and mutant clones by Phyre 2.0 software. Both *Pdpn*–KD/TC and *Pdpn*–KO clones showed altered protein structures with missing alpha helix structure at N–terminal region, indicating loss of function. Rainbow color gradient: red indicates the N–terminal while blue indicates C–terminal of protein. **(D)** Immunoblot verification of Pdpn protein level in mutant clones. Stable clones were established following CRISPR/Cas9 editing. Pdpn protein was induced after 24 hours stimulation with *H. pylor*i at an MOI of 10. Pdpn induction were suppressed at ~70% and > 99% in the two selected clones, *Pdpn*–KD/TC and *Pdpn*–KO, respectively. Anti–Pdpn was used for detection and β–actin as loading control. Asterisk (*) represents truncated form was detected in *Pdpn*–KD/TC. Ladder indicates molecular weight, kDa: kiloDalton.

**
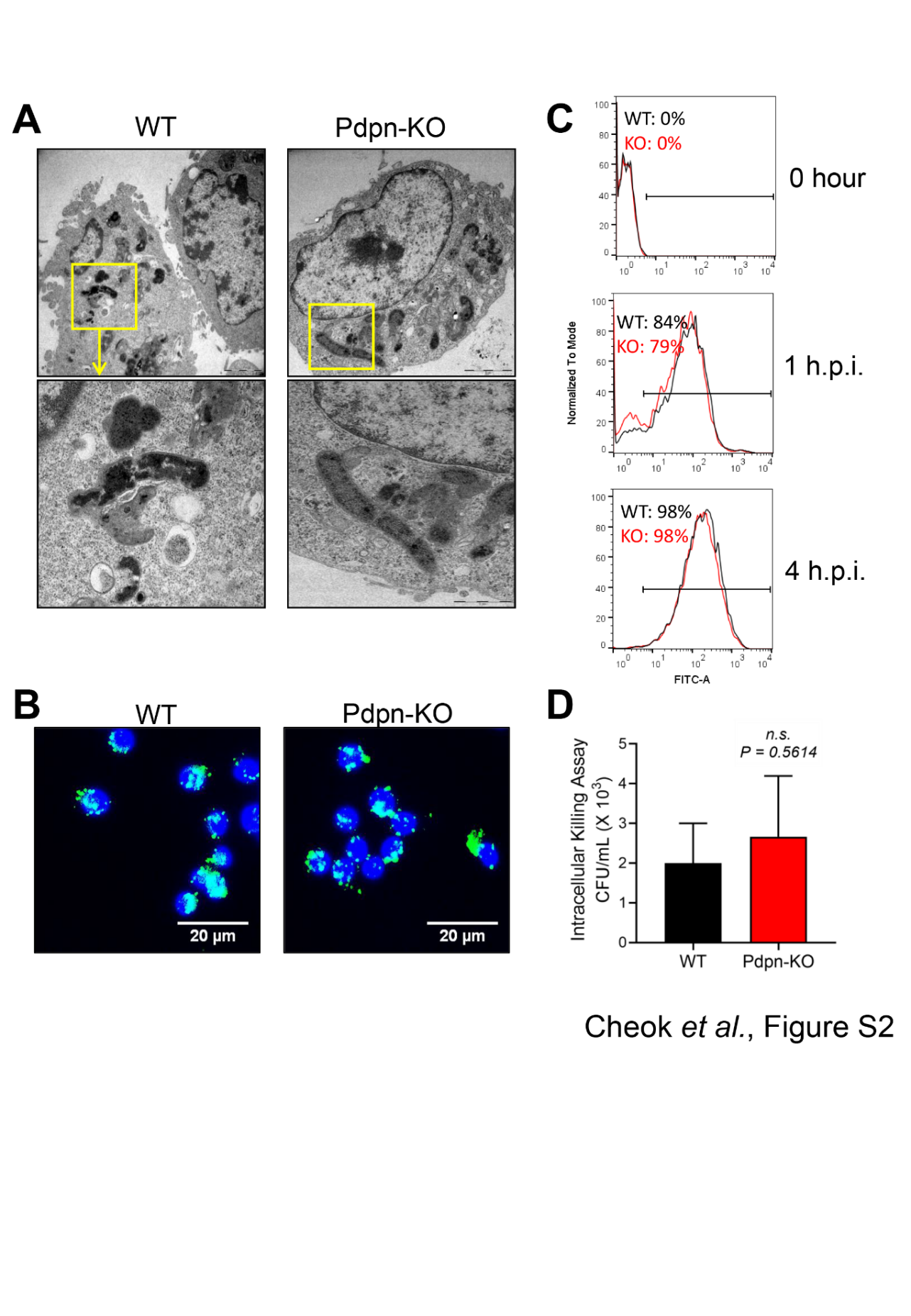
**

**Supplementary Figure S2. Phagocytosis and bactericidal activities in *Pdpn***–**KO cells.**

**(A)** Representative images from transmission electron microscope. Wildtype (WT) or *Pdpn*–KO cells were infected with *H. pylori* SS1 at MOI 10 for 24 hours before fixation. Curved bacillary, dense structures represent *H. pylori* internalized into cell phagosomes as indicated. Scale bars represent 2 µm, or 500 nm after magnification. **(B)** Internalization of FITC–labelled *H. pylori*. Flow cytometry histogram indicates FITC intensities of wildtype (WT) and *Pdpn*–KO after bacteria uptake, before (mock) and after *H. pylori* infection (MOI 1) for 1 or 4 hours. Percentages of FITC–positive cells in the gates applied were as indicated. Red lines represent *Pdpn*–KO; black line represent WT cells. **(C)** Representative immunofluorescent images of *H. pylori* (MOI 10, 24 hours) infected–wildtype and *Pdpn*–KO cells. Bacterial cells were labelled with FITC (green), nucleus was stained with DAPI (blue), and viewed under a fluorescent microscope. Scale bars indicate 20 µm. **(D)** Intracellular killing assay. Bar chart represents the number of viable bacterial count after 24 hours incubation of *H. pylori* SS1 at MOI 10 in the wildtype (WT) or *Pdpn*–KO cells. Data are shown as mean ± *SD* representative of two independent experiments. *n.s.:* not significant by Student’s *t*–test.

**
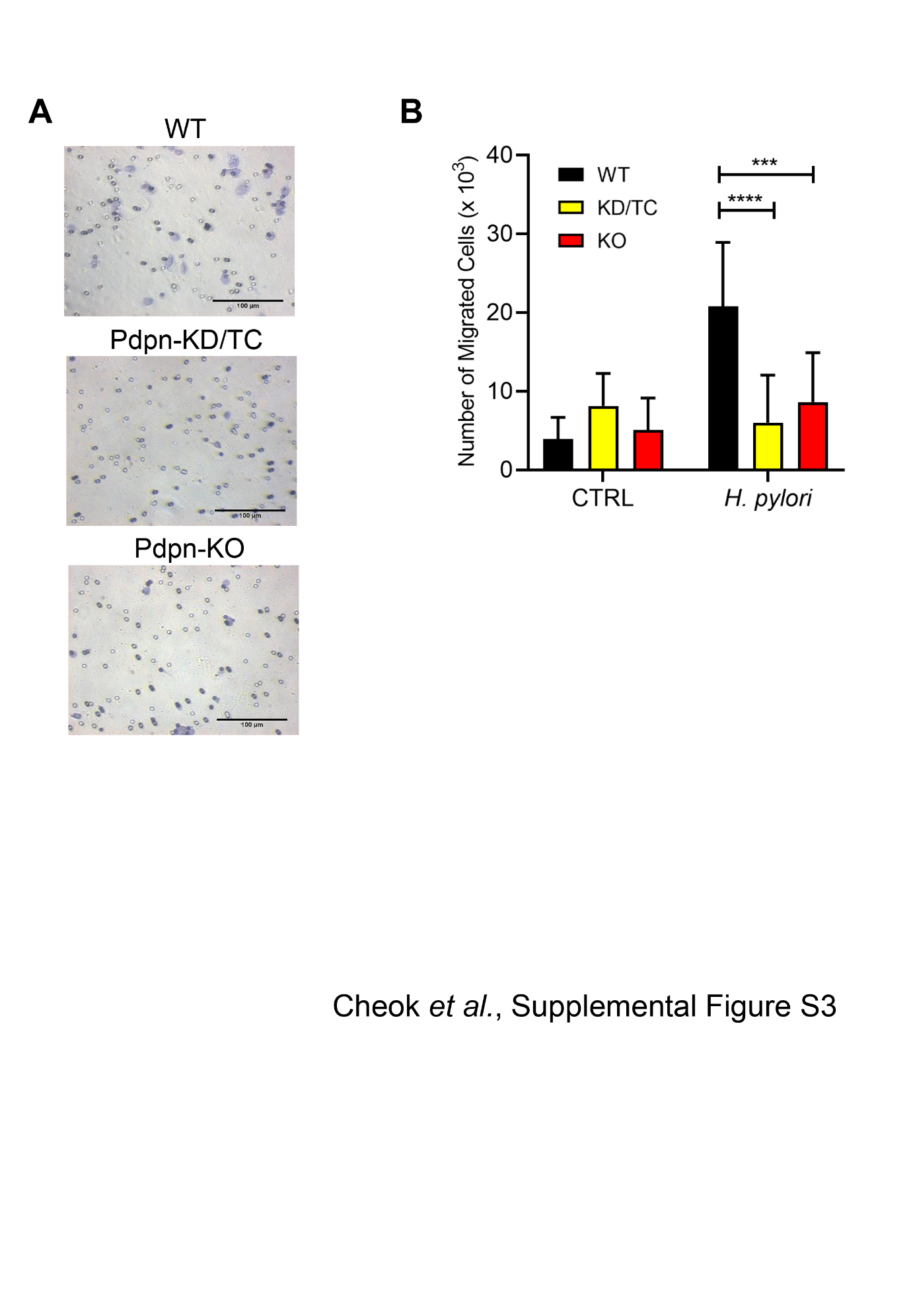
**

**Supplementary Figure S3. Migration assay using heat-inactivated *H. pylori*.**

**(A)** Representative images of migrated cells for wildtype (WT), Pdpn–KD/TC and Pdpn–KO cells in transwell migration assay. Cells seeded at upper chamber of 8 μm transwell insert were induced with heat-inactivated *H. pylori* (MOI 50:1) from the lower chamber and processed with crystal violet staining after 24 hours. Circles indicate the pores in the transwell. Scale bars indicate 100 μm. **(B)** Histogram shows number of migrated cells in transwell migration assay. Data was shown as mean ± SD from eight randomly selected fields of the transwell. Statistical significance was analyzed with two–way ANOVA test (F value: 12.48; Degree of Freedom: 42; ***P < 0.001, ****P < 0.0001).

**
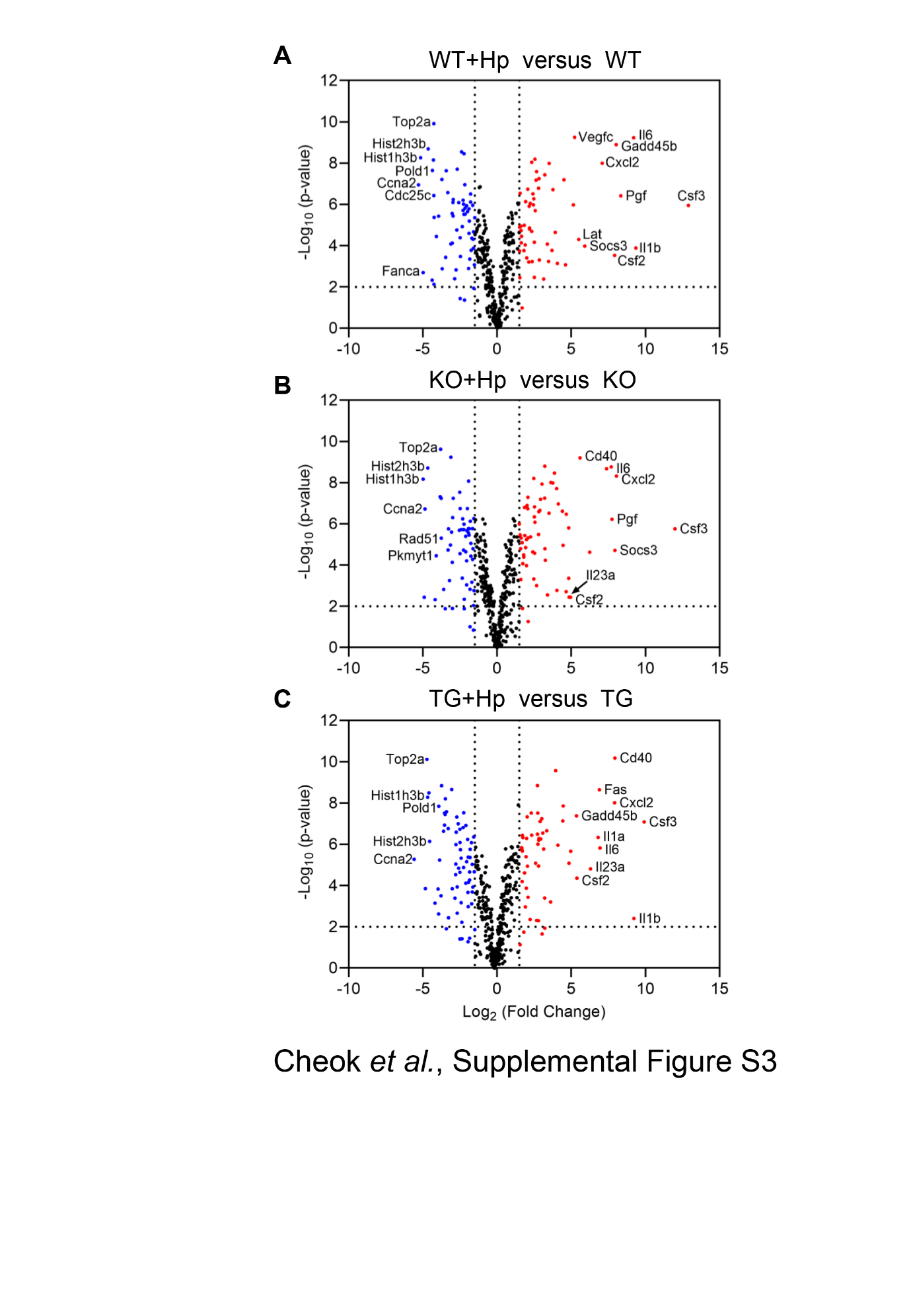
Supplementary Figure S4. Volcano plots for comparison between *H. pylori*–infected versus uninfected cells.**

Volcano plots show the comparison between *H. pylori*–infected versus uninfected **(A)** WT, **(B)** *Pdpn*–KO and **(C)** *Pdpn*–TG groups. Vertically broken lines demarcate log_2_ (fold change) at > 1.5 or < -1.5. Horizontally broken lines demarcate statistical significance (-log_10_ *P*–value > 2, or *P* < 0.01) by R–based program. Significantly upregulated and downregulated genes were highlighted in red and blue dots, respectively.

**
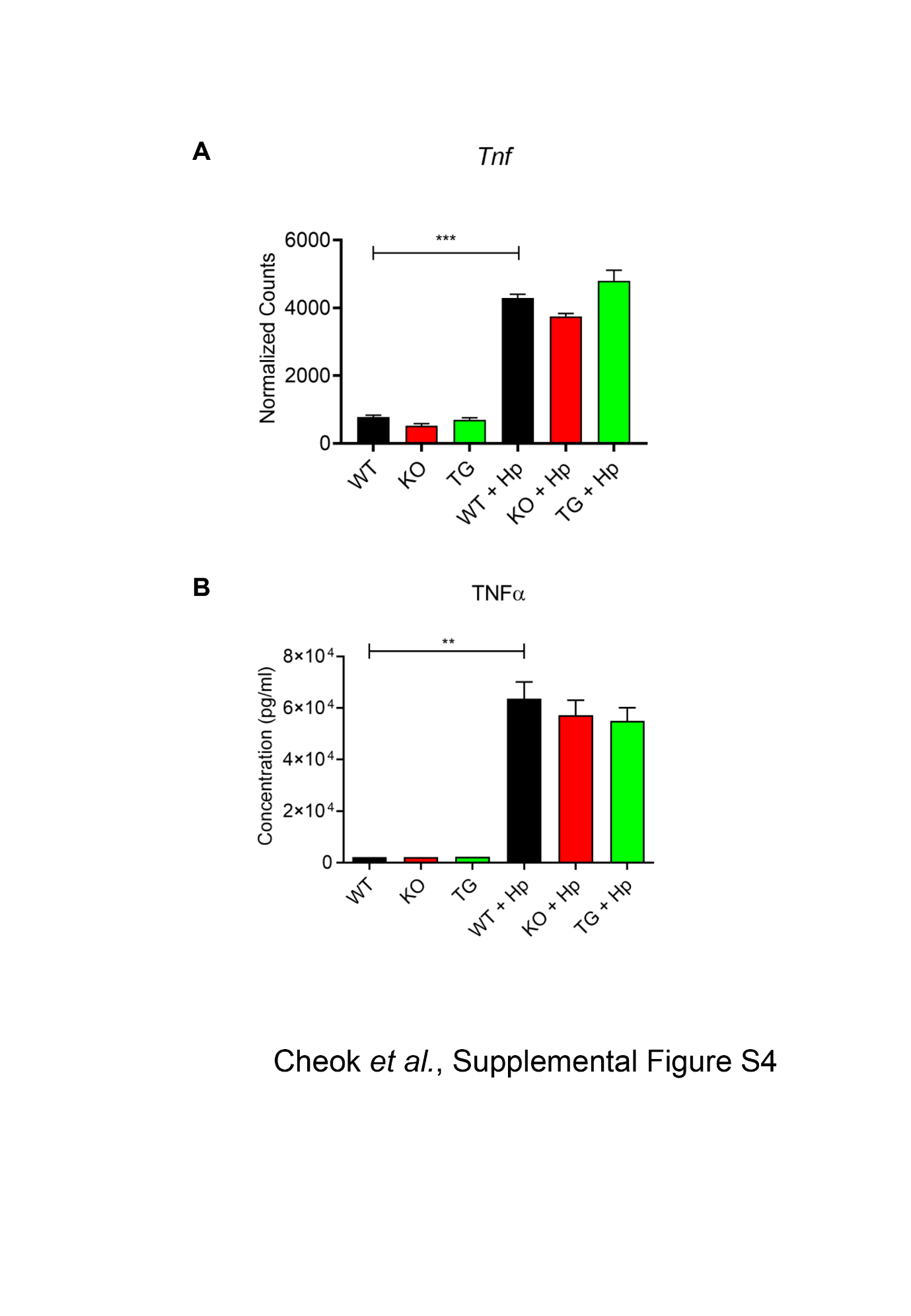
Supplementary Figure S5. TNFα protein secretion was unaffected in Pdpn expression.**

Wildtype (WT), *Pdpn*–knockout (KO) and *Pdpn*–transgenic (TG) cells, before and after *H. pylori* infection at MOI 10 for 24 hours (as indicated by +Hp). **(A)** Bar chart show normalized RNA transcript counts of *Tnf* from NanoString analysis. Data are shown as mean ± *SD* of duplicate samples. Statistical significance was analyzed with unpaired Student’s *t*–test (****P* ≤ 0.001). **(B)** Bar chart show ELISA analysis of TNFα production in cell lysate. Y axis represents cytokine concentrations in pg/ml. Data were shown as mean ± *SD* of duplicates obtained from representative of two independent experiments. Statistical significance by unpaired Student’s *t*–test (***P* < 0.01).

# Supplementary Video

**Supplementary Video S1. Disrupted cell migration capability following *Pdpn*–deletion.**

Wildtype and *Pdpn*–KO cells were seeded at > 90% confluency in 96 well plate. A scratch was subsequently introduced, and cell wound healing ability for both cells was recorded concurrently by NanoEntek JuLi BRD04 live cell imager every 5 minutes for 24 hours. Note that decreased ability of *Pdpn*–KO in migrating to the scratched area of lesser cell density compared to wildtype in would healing assay. *Pdpn*–KO appeared round morphologically, demonstrated limited motility and less formation of filopodia–like structures, when compared to wildtype cells. Data shown is representative of three independent experiments.
